# The Protective Effect of Theaflavins on the Kidney of Mice with Type II Diabetes Mellitus

**DOI:** 10.3390/nu15010201

**Published:** 2022-12-31

**Authors:** Jun Wang, Jingjing Jiang, Changyu Zhao, Hongyan Shan, Ziheng Shao, Chun Wang, Jiayun Guan, Zhongwen Xie, Songnan Li

**Affiliations:** 1 School of Tourism and Cuisine, Yangzhou University, Yangzhou 225127, China; 2 State Key Laboratory of Tea Plant Biology and Utilization, Anhui Agricultural University, 130 Changjiang West Road, Hefei 230036, China; 3 Joint International Research Laboratory of Agriculture and Agri-Product Safety of the Ministry of Education of China, Institutes of Agricultural Science and Technology Development, Yangzhou University, Yangzhou 225009, China

**Keywords:** diabetes, theaflavins, AGEs, RAGE, MAPK, NF-κB

## Abstract

Diabetic nephropathy, primarily caused by advanced glycation end products (AGEs), is a serious complication resulting from type 2 diabetes mellitus (T2DM). Reportedly, theaflavins (TFs) can improve diabetic nephropathy; however, the underlying molecular mechanism is not fully clear. In this study, T2DM mice were treated with different concentrations of TFs by gavage for 10 weeks to investigate the effect of TFs on diabetic nephropathy and their potential molecular mechanism of action. Biochemical and pathological analysis showed that the TFs effectively improved blood glucose, insulin resistance, kidney function, and other symptoms in diabetic mice. The mechanism studies indicated that TFs inhibited the formation of AGEs, thereby inhibiting the activation of the MAPK/NF-κB signaling pathway. Therefore, our study suggested that TFs improved diabetic nephropathy by inhibiting the formation of AGEs.

## 1. Introduction

Persistent hyperglycemia in patients with diabetes causes various complications, including diabetic nephropathy, retinopathy, osteoporosis, and others [1,2]. Diabetic nephropathy, one of the most common and serious complications of diabetes, has become the leading cause of end-stage renal disease in most countries [3]. Therefore, the prevention or treatment of diabetic nephropathy is a major area of research in clinical science. However, several synthetic drugs used in clinical practice to prevent and treat diabetic nephropathy exert multiple adverse effects in patients, such as hyperkalemia, hypotension, and renal dysfunction [4,5]. Theaflavins (TFs) are flavonoid compounds formed by the oxidative condensation of catechins or their derivatives in tea [6]. Findings from recent studies have demonstrated that TFs not only protect kidneys through antioxidant, anti-inflammatory, and anticancer pharmacological effects [6,7,8,9] but also improve T2DM, hypertension, and hyperlipidemia caused by obesity [10]. Meanwhile, Sarkar et al. [11] showed that black tea extract rich in TFs exhibits a protective role against diabetic nephropathy in rats. However, even though the protective effects of TFs against diabetic nephropathy have been reported, the intrinsic molecular mechanism of action of theaflavins in diabetic nephropathy is not fully clear. In this article, we focus on its renoprotective effects in diabetic mice, and explore the intrinsic molecular mechanism.

Advanced glycation end products (AGEs) are key factors in the development of diabetic nephropathy and have been studied extensively in recent years. Initially, they are produced as stable compounds by spontaneous non-enzymatic glycosylation reactions between reducing sugars and proteins [3]. Exogenous AGEs are mostly formed in food processing, primarily from the Maillard reaction. Endogenous AGEs are usually formed from glycolysis in vivo and can accumulate in tissues [12]. Long-term hyperglycemia and oxidative stress in patients with T2DM produce a large number of reactive carbonyl compounds, such as glyoxal (GO), methylglyoxal (MGO), malonaldehyde (MDA), and others. These compounds irreversibly form various AGEs, such as pentosidine, Nε-carboxymethyl lysine (CML), and Nε-carboxyethyl lysine (CEL), through a series of reactions with the terminal amino groups of macromolecules such as proteins, lipids, or nucleic acids [13]. Once formed, these AGEs accumulate in the kidneys with age [14], and eventually lead to nephropathy [2]. The destructive effects of AGEs on the kidneys are primarily mediated through two pathways: the non-receptor pathway and the receptor pathway. In the non-receptor pathway, AGEs change the structure of collagen, laminin, and some other persistent proteins in the glomerular thylakoid and basement membranes, thus accelerating kidney tissue damage [15]. The receptor pathway is the most important pathway by which AGEs act on kidney cells. AGEs bind to receptors for advanced glycation end products (RAGEs) and activate certain signaling pathways, such as mitogen-activated protein kinase (MAPK)/NF-κB, JAK-STAT, and PI3K/Akt/mTOR, which are involved in inflammation, fibrosis, apoptosis, and other processes and eventually damage the structure and function of the kidneys [3,16,17]. Therefore, suppressing AGEs formation or blocking signal transduction induced by AGEs is essential for preventing and treating diabetic nephropathy.

TFs may protect the kidneys of diabetic mice by blocking the MAPK/NF-κB signaling cascade. Catechins, which are TFs precursors, can improve diabetic nephropathy by scavenging MGO in vivo to prevent AGEs formation [18,19,20]. The four major monomers, theaflavin, theaflavin-3-gallate, theaflavin-3′-gallate, and theaflavin-3,3′-digallate, can all scavenge MGO in vitro [6]. Theaflavin-3,3′-digallate scavenges approximately twice as much MGO as catechins [21,22]. Thus, TFs may protect the kidney by inhibiting AGEs formation by reducing the levels of reactive carbonyl compounds, such as MGO and MDA. Additionally, TFs were shown to reduce inflammation by inhibiting the activation of the MAPK/NF-κB signaling pathway [23,24,25,26,27]. Considering the relationship among AGEs, the MAPK/NF-κB signaling pathway, and the effects of TFs on the kidney (described above), TFs may block the MAPK/NF-κB signaling cascade by reducing the level of AGEs in vivo to improve diabetic nephropathy. In this study, T2DM mice, fed a high-fat diet and administered streptozotocin, were treated with different doses of TFs to investigate the renoprotective effects and the underlying molecular mechanism of action of TFs in diabetic mice.

## 2. Materials and Methods

### 2.1. Materials

TFs (>80% purity) were purchased from Dehe Bio-Technology Co., Ltd. (Jiangsu, Wuxi, China).

### 2.2. Animals

In this study, the mice used were six-week-old male C57BL/6 mice. Ten mice were fed a basic diet (5% fat) and 40 mice were fed a high-fat diet (60% fat) for 4 weeks. Following this, the mice fed a high-fat diet were injected with streptozotocin (40 mg/kg body weight/day), and the mice fed the basic diet were injected with an equal volume of sterile sodium citrate buffer for 5 consecutive days [28]. In the 12th week, mice with fasting blood glucose levels greater than 11.1 mmol/L were diagnosed with T2DM [29] and fed a high-fat diet for another 8 weeks to induce diabetic nephropathy. Subsequently, T2DM mice were randomly divided into four groups: diabetic model control (MC), low-dose TFs (LTFs), high-dose TFs (HTFs), and metformin (Met) (positive control). The mice fed the basic diet were in the normal control (NC) group. These mice were treated daily for 10 weeks with the following administered orally: (1) NC group, normal saline; (2) MC, normal saline; (3) LTFs, 50 mg/kg body weight/day TFs; (4) HTFs, 150 mg/kg body weight/day TFs; and (5) Met, 150 mg/kg body weight/day metformin.

### 2.3. Oral Glucose Tolerance Test (OGTT) and Insulin Tolerance Test (ITT)

An OGTT and an ITT were performed in all mice separately after 10 weeks of intervention. Glucose (0.5 g/kg body weight) was orally administered to mice that were fasted for 16 h. Insulin (1 U/kg body weight) was injected in mice that were fasted for 6 h. Blood glucose levels were measured at 0, 15, 30, 60, and 120 min after administration.

### 2.4. ELISA

The CML, CEL, MGO, insulin, and NADPH oxidase activity in the plasma or kidneys were measured using ELISA kits (Sinobest, Shanghai, China) according to the manufacturer’s instructions. The homeostasis model assessment of insulin resistance (HOMA-IR) and insulin sensitivity (HOMA-IS) were analyzed according to methods reported in the literature [30].

### 2.5. Biochemical Analysis

The levels of glycosylated hemoglobin type A1C (HbA1c), 24 h urinary protein, urinary albumin, urine creatinine (UCr), blood urea nitrogen (BUN), serum creatinine (SCr), and malonaldehyde (MDA) were tested using kits (Jiancheng, Nanjing, China). The glomerular filtration rate (GFR) was calculated according to a reported protocol [31].

### 2.6. Histological Examination

Kidney tissues were immersed in 4% paraformaldehyde for more than 48 h. Routine paraffin slices were prepared and stained with hematoxylin and eosin (HE) for histological assessment using light microscopy.

### 2.7. Total AGEs Assay

The total AGEs in plasma or kidney tissues were estimated using a previously reported method [20,32]. The fluorescence intensity was expressed in AU normalized by the reading from 1 mg/mL of BSA.

### 2.8. mRNA Extraction and Quantitative Real-Time-PCR (qPCR) Analysis

Total mRNA was extracted from kidney tissues for qPCR analysis using the SYBR Green kit according to the instructions (Takara, Beijing, China). The primers used in the experiment are *Gapdh* (F: 5′-ATCAACGGGAAGCCCATCAC-3′; R: 5′-TTGGCTCCACCCTTCAAGTG-3′); *Rage* (F: 5′-GACCCTTAGCTGGCACTTGG-3′; R: 5′-AGCGTGAAGAGTCCCGTCTC-3′); *Tnfα* (F: 5′-TGATCGGTCCCAACAAGGA-3′; R: 5′-TGCTTGGTGGTTTGCTACGA-3′); *Il1β* (F: 5′-TCGCAGCAGCACATCAACAAGAG-3′; R: 5′-TGCTCATGTCCTCATCCTGGAAGG-3′); *Il6* (F: 5′-CCAGTTGCCTTCTTGGGACT-3′; R: 5′-GGTCTGTTGGGAGTGGTATCC-3′); *Tgfβ1* (F: 5′-CTGTGGAGCAACACGTAGAACTCT-3′; R: 5′-TGTATTCCGTCTCCTTGGTTCA-3′); fibronectin (F: 5′-CACCAACGAACTTGCACCTG-3′; R: 5′-CTGATCGGCATGGACCACTT-3′).

### 2.9. Western Blot Analysis

Total proteins were extracted from kidney tissues using the RIPA lysis buffer. Western blot analysis was performed with standard procedures using antibodies against the following proteins: RAGE, p65, p-p65, p38, p-p38, ERK1/2, p-ERK1/2, TGF-β1, TNF-α, and GAPDH (Cell Signaling, Danvers, MA, USA).

### 2.10. Statistical Analysis

Data are presented as mean ± standard error of mean (SEM). Graphs were prepared with the statistical data using GraphPad Prism 8.0 (Graph Pad, La Jolla, CA, USA). The differences between groups were analyzed using one-way ANOVA. *p* < 0.05 was considered significant.

## 3. Results

### 3.1. Effects of TFs on Fasting Blood Glucose, Body Weight, Food Consumption, Water Consumption, and Urine Volume in T2DM Mice

**Figure 1:**
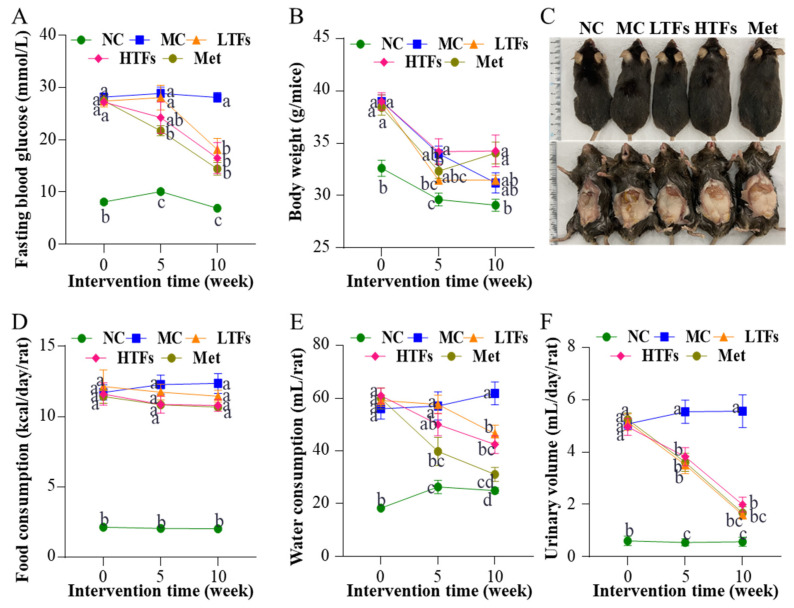
Changes in fasting blood glucose, body weight, food consumption, water consumption, and urinary volume in T2DM mice. Changes in fasting blood glucose (**A**), body weight (**B**), body and abdominal fat (**C**), food consumption (**D**), water consumption (**E**), and urinary volume (**F**). NC: normal control; MC: diabetic model control; LTFs: low-dose TFs; HTFs: high-dose TFs; Met: metformin. Each value represents the mean ± SEM (*n* = 10). Values at the same column with different letters (a–d) differ from each other significantly (*p* < 0.05).

After 20 weeks of high-fat feeding (week 0 of the intervention experiment), the fasting blood glucose level of mice fed the high-fat diet was greater than 11.1 mmol/L (Figure 1A). The body weight (Figure 1B) (*p* < 0.0001), food consumption (Figure 1D) (*p* < 0.0001), water consumption (Figure 1E) (*p* < 0.0001), and urinary volume (Figure 1F) (*p* < 0.0001) were significantly higher than those of normal controls (NC). These typical symptoms of diabetes mellitus indicated the successful establishment of T2DM mouse models. During the 10-week intervention with TFs or metformin, the fasting blood glucose level in the MC mice was maintained at 28.0 mmol/L. In contrast, mice from the LTFs, HTFs, and Met groups all had significantly lower fasting blood glucose levels after 10 weeks of intervention (LTFs vs. MC, *p* < 0.0001; HTFs vs. MC, *p* < 0.0001; Met vs. MC, *p* < 0.0001) (Figure 1A). The fasting blood glucose levels of LTFs and HTFs were all similar with that of Met mice. The data demonstrated that TFs, similar to metformin, can effectively improve the hyperglycemic symptoms of T2DM mice. During the intervention, as the mice developed diabetes mellitus, the body weight of MC mice decreased persistently from 39 g to 31.2 g. The rate of weight loss in each group indicated that during the first 5 weeks of the intervention, all T2DM mice lost weight compared to NC mice (rate of weight loss: MC: 1.0 g/week; LTFs: 1.3 g/week; HTFs: 1.0 g/week; Met: 1.2 g/week). However, as the duration of intervention increased, the rate of weight loss in mice in the TFs and metformin intervention groups slowed down significantly compared with that in the MC group (rate of weight loss during the last 5 weeks of intervention: MC: 0.48 g/week; LTFs: −0.00040 g/week; HTFs: −0.098 g/week; Met: −0.19 g/week). At the end of the intervention (week 10), the weights of mice from the HTFs and Met groups were 9.83% and 9.19% greater than those of mice from the MC group (Figure 1B). And there was no difference for the weights between HTFs and Met mice. These changes in weight and the images of the body and abdominal fat of mice (Figure 1C) confirmed that TFs could effectively prevent weight loss in T2DM mice just like metformin. No significant changes were observed in the food intake of T2DM mice before and after TFs or metformin intervention (week 10 of the intervention experiment: LTFs vs. MC, *p* = 0.83; HTFs vs. MC, *p* = 0.38; Met vs. MC, *p* = 0.39) (Figure 1D). Thus, the inhibitory effects of TFs and metformin on body weight loss in T2DM mice were not caused by the change in food consumption. However, the water consumption (Figure 1E) and urinary volume (Figure 1F) of the LTFs, HTFs and Met mice decreased significantly compared to those of MC mice. Except for the water consumption of LTFs mice, these data of LTFs and HTFs mice was all similar to those of Met mice. These data suggested that TFs could effectively alleviate hyperglycemia, weight loss, polydipsia, and polyuria in T2DM mice but did not affect their dietary intake.

### 3.2. Effects of TFs on Glucose and Insulin Homeostasis in T2DM Mice

**Figure 2:**
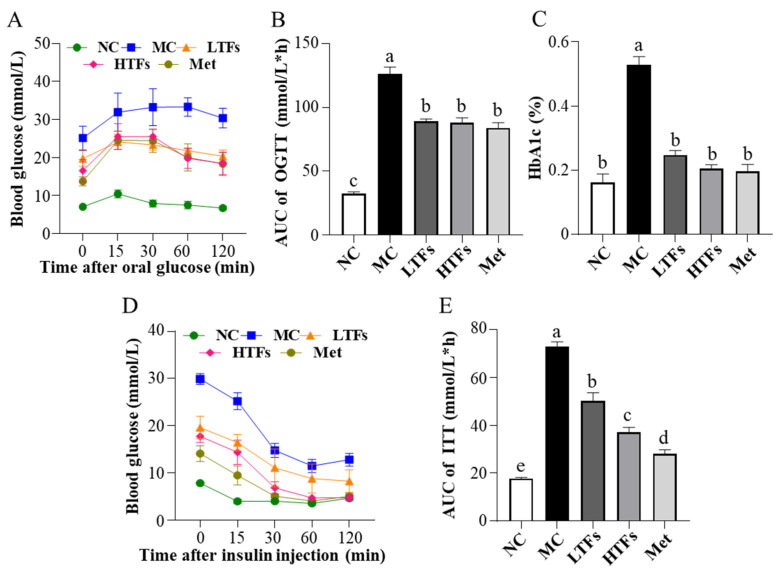
Effects of TFs on blood glucose and insulin homeostasis in T2DM mice. (**A**) Oral glucose tolerance test (OGTT) levels; (**B**) area under the curve (AUC) values of OGTT; (**C**) glycosylated hemoglobin type A1C (HbA1c) levels; (**D**) insulin tolerance test (ITT) levels; (**E**) area under the curve (AUC) values of ITT. NC: normal control; MC: diabetic model control; LTFs: low-dose TFs; HTFs: high-dose TFs; Met: metformin. Each value represents the mean ± SEM (*n* = 6). Values with different letters (a–e) differ from each other significantly (*p* < 0.05).

Glucose and insulin homeostasis are disrupted, and glucose and insulin tolerances are impaired with the development of T2DM. To evaluate the regulatory effects of TFs on glucose and insulin homeostasis in T2DM mice, glucose tolerance, insulin tolerance, and the HbA1c and insulin levels were examined in each group at the end of the intervention. The results of the OGTT showed that after the oral administration of glucose solution, the blood glucose levels of MC mice remained greater than 30.0 mmol/L until 60 min. Thus, their glucose tolerance was severely impaired (Figure 2A). In contrast, mice in the LTFs, HTFs, and Met groups had significantly lower blood glucose levels than those in the MC group, and the level decreased after peaking at 15 min (Figure 2A). The area under the curve (AUC) based on the blood glucose level at each time point was significantly lower in all intervention groups compared to that in the MC group (LTFs vs. MC, *p* < 0.0001; HTFs vs. MC, *p* < 0.0001; Met vs. MC, *p* < 0.0001) (Figure 2B). The difference between the LTFs and Met mice was not significant, nor the difference between the HTFs and Met mice. This confirmed the changes in glucose tolerance. As one of the diagnostic criteria of diabetes, the HbA1c level indicates long-term changes in blood glucose in mice. At the end of the experiment, the HbA1c level of MC mice was 3.24 times higher than that of NC mice (*p* < 0.0001). After the intervention with TFs or metformin, the HbA1c levels of LTFs, HTFs, and Met mice were significantly lower than that of MC mice (LTFs vs. MC, *p* < 0.0001; HTFs vs. MC, *p* < 0.0001; Met vs. MC, *p* < 0.0001) (Figure 2C). When comparing the data from LTFs and HTFs mice with Met mice, no significant differences were found. These results suggested that TFs improved the glucose homeostasis in T2DM mice.

Insulin regulates blood glucose levels. However, insulin homeostasis is disrupted in T2DM mice. The high level of blood glucose after insulin injection in the MC mice within the ITT suggested that the insulin tolerance of these mice was impaired (Figure 2D), while the blood glucose level at each time point in the ITT curve decreased significantly after TFs intervention (Figure 2D). The AUCs of the LTFs, HTFs, and Met groups decreased by 30.99%, 48.98%, and 61.33%, respectively, compared with that in the MC group (LTFs vs. MC, *p* < 0.0001; HTFs vs. MC, *p* < 0.0001; Met vs. MC, *p* < 0.0001) (Figure 2E). The AUCs of LTFs and HTFs groups were all significantly lower than that of Met group. This indicated that TFs could significantly improve the impairment of insulin tolerance in T2DM mice in a dose-dependent manner. The insulin homeostasis model is widely used to assess insulin resistance and insulin sensitivity. At the end of the experiment, the plasma insulin levels were 2.41 times higher in the MC group than in the NC group (*p* < 0.05) (Table 1). However, the levels reduced significantly in response to TFs intervention (LTFs vs. MC, *p* = 0.0044; HTFs vs. MC, *p* < 0.0001) (Table 1). The 9.90-fold increase in the HOMA-IR index in the MC group compared with that in the NC group (*p* < 0.0001) suggested insulin resistance in MC mice (Table 1). The HOMA-IR indices of the LTFs, HTFs, and Met groups decreased by 46.08%, 64.18%, and 79.84%, respectively, compared with that in the MC group (LTFs vs. MC, *p* = 0.0005; HTFs vs. MC, *p* < 0.0001; Met vs. MC, *p* < 0.0001) (Table 1). This indicated that TFs could improve insulin resistance in T2DM mice in a dose-dependent manner, which was consistent with the results of the ITT experiment. The HOMA-IS index indicated that the insulin sensitivity in MC mice was only approximately 10% of that in NC mice (*p* < 0.0001) (Table 1). After 10 weeks of intervention, the HOMA-IS was significantly higher in both HTFs and Met groups compared with that in the MC group (LTFs vs. MC, *p* = 0.57; HTFs vs. MC, *p* < 0.035; Met vs. MC, *p* < 0.0001) (Table 1). Thus, the insulin sensitivity of T2DM mice could be restored by TFs intervention. In conclusion, TFs could improve glucose and insulin homeostasis in T2DM mice. The insulin levels and HOM-IR of HTFs mice and Met mice suggested that high-dose TFs had the equal positive effect as metformin in regulating the glucose and insulin homeostasis in T2DM mice.

### 3.3. TFs Intervention Improved Kidney Function and Structure in T2DM Mice

**Figure 3:**
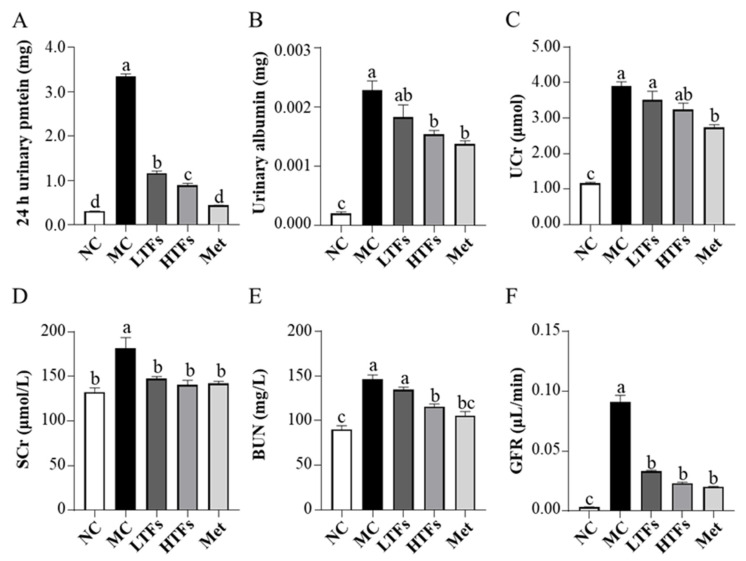
Effects of TFs on kidney function in T2DM mice. (**A**) 24 h urinary protein levels; (**B**) urinary albumin levels; (**C**) urine creatinine (UCr) levels; (**D**) serum creatinine (SCr) levels; (**E**) blood urea nitrogen (BUN) levels; (**F**) glomerular filtration rate (GFR) levels. NC: normal control; MC: diabetic model control; LTFs: low-dose TFs; HTFs: high-dose TFs; Met: metformin. Each value represents the mean ± SEM (*n* = 6). Values with different letters (a–d) differ from each other significantly (*p* < 0.05).

**Figure 4:**
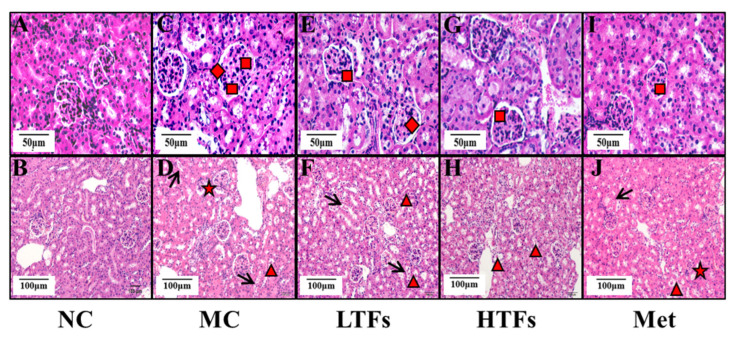
The HE-stain images of kidney tissues of the mice in each group. (**A**,**B**): kidney tissues images of normal control group mice. (**C**,**D**): kidney tissues images of diabetic model control group mice. (**E**,**F**): kidney tissues images of low-dose TFs group mice. (**G**,**H**): kidney tissues images of high-dose TFs group mice. (**I**,**J**): kidney tissues images of metformin group mice. Square: glomerular mesangial expansion; rhombus: hyalinization of renal glomerular; triangle: renal tubular epithelial cell exfoliation; arrow: renal tubular epithelial cell vacuolar degeneration; star: glomerular atrophy. Scale bars are 50 μm and 100 μm respectively. NC: normal control; MC: diabetic model control; LTFs: low-dose TFs; HTFs: high-dose TFs; Met: metformin.

The major clinical manifestations of DN include increased urinary protein, urinary microalbumin excretion, and a greater glomerular filtration rate. At the end of the experiment, the 24 h urinary protein (Figure 3A) and urinary microalbumin (Figure 3B) levels in the MC group were 10.94 (*p* < 0.0001) and 11.23 (*p* < 0.0001) times greater than those in the NC group, respectively. After TFs or metformin intervention, the levels of these two indicators decreased significantly (Figure 3A,B). Compared with that in the MC group, the urinary microalbumin levels in the HTFs group decreased by 32.72%. This change was similar to that observed in the Met group (LTFs vs. MC, *p* = 0.14; HTFs vs. MC, *p* = 0.011; Met vs. MC, *p* = 0.0028) (Figure 3B). Creatinine and urea nitrogen are key indicators of the glomerular filtration capacity. Compared with that in NC mice, the levels of UCr (Figure 3C), SCr (Figure 3D), and BUN (Figure 3E) in MC mice were elevated by 235.95% (*p* < 0.0001), 36.99% (*p* = 0.000), and 62.71% (*p* < 0.0001), respectively. This indicated the impaired kidney function of T2DM mice. However, the SCr (Figure 3D) and BUN (Figure 3E) levels in the HTFs mice decreased by 22.53% (*p* = 0.0027) and 21.16% (*p* = 0.0003), respectively. Similar changes were observed in Met mice. At the end of the experiment, GFR, the essential indicator of diabetic nephropathy in MC mice, was 27 times greater than that in NC mice (NC vs. MC, *p* < 0.0001) (Figure 3F). After 10 weeks of treatment, the GFR of mice in each intervention group decreased by more than 60% (LTFs vs. MC, *p* < 0.0001; HTFs vs. MC, *p* < 0.0001; Met vs. MC, *p* < 0.0001) (Figure 3F). When comparing the data from HTFs mice with that of Met mice, except for the 24 h urinary protein, no significant differences were found. These changes indicate that TFs can improve the kidney function of T2DM mice, and the high dose of TFs had an equally positive effect when compared to Met. Besides the changes in the kidney function, the kidney tissues of T2DM mice with diabetic nephropathy showed structural changes, including glomerular mesangial expansion, hyalinization of renal glomerular, glomerular atrophy, tubular and vascular lesions, and other symptoms [4]. HE-stained images of the kidney tissues of MC mice showed obvious pathological features of diabetic nephropathy, such as glomerular mesangial expansion, hyalinization of renal glomerular, and glomerular atrophy, among others (Figure 4C,D). After the TFs or metformin intervention, these characteristic pathological changes of diabetic nephropathy improved significantly (Figure 4E–J).

### 3.4. TFs Intervention Reduced the Level of AGEs in T2DM Mice

In patients with diabetes, glucose, present in abundance, is the major source of carbonyl compounds such as MGO, GO, and MDA, which are precursors of AGEs. Therefore, compared with healthy individuals, patients with diabetes are more likely to have a higher level of AGEs [2]. Recent studies have shown that AGEs accumulating in the kidneys of patients with diabetes play an important role in diabetic nephropathy development [3]. In this study, compared with that in NC mice, the level of total plasma AGEs in MC mice was 41.64% higher (*p* = 0.0068), and the level of total kidney AGEs was 232.08% higher (*p* < 0.0001) (Table 2). After the high-dose TFs intervention, the total AGE level of HTFs mice decreased by 27.44% (*p* = 0.012) in the plasma and 26.43% (*p* = 0.042) in the kidneys (Table 2). The same trend was observed in Met mice. CML and CEL, which are biomarkers for the assessment of a diabetic condition [33], have been confirmed to be signature ligands for RAGE [34]. As shown in Table 2, the plasma levels of CML and CEL in MC mice were elevated by 21.75% (*p* = 0.033) and 22.79% (*p* = 0.0016), respectively, compared to those in NC mice, which is similar to the findings of O′Grady et al. [35]. After high-dose TFs or metformin intervention, the level of both CML and CEL in plasma or kidney tissues was reduced significantly compared with that in MC mice. Compared with Met mice, the levels of total plasma AGEs and total kidney AGEs in LTFs or HTFs mice showed no significant difference (Table 2). The levels of plasm CML, plasm CML and kidney CEL in LTFs mice but HTFs mice were higher than those in Met mice (Table 2). The above results suggest that TFs can effectively reduce the level of AGEs in T2DM mice. MGO and MDA are the primary precursors of AGEs [3,15], and their contents directly affect the level of AGEs. As shown in Table 2, the plasma and kidney levels of MGO and MDA in the five groups of mice changed similarly to the levels of AGEs. Thus, TFs might inhibit AGEs formation by reducing the levels of MGO and MDA, the precursors of AGEs.

### 3.5. TFs Improved Inflammation and Fibrosis in the Kidney of T2DM Mice

**Figure 5:**
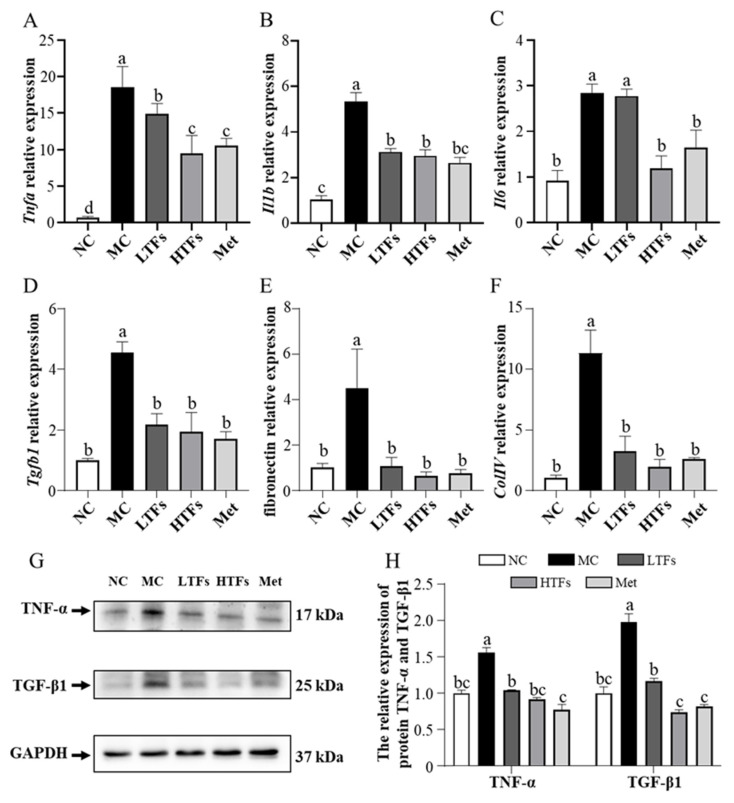
Inflammation and fibrosis in the kidney tissues of T2DM mice. The mRNA expression of inflammatory markers: *Tnfa* (**A**), *Il1b* (**B**), and *Il6* (**C**); the mRNA expression of fibrosis markers: *Tgfb1* (**D**), fibronectin (**E**), and *ColIV* (**F**); (**G**) Western blot analysis of TNF-α and TGF-β1; (**H**) quantification of TNF-α and TGF-β1 protein expression. NC: normal control; MC: diabetic model control; LTFs: low-dose TFs; HTFs: high-dose TFs; Met: metformin. Each value represents the mean ± SEM (*n* = 6). Values with different letters (a–c) differ from each other significantly (*p* < 0.05).

AGEs bind to RAGE and then activate the MAPK/NF-κB pathway, which is involved in inflammation and fibrosis, and eventually destroy the structure and function of the kidneys [3]. To verify the changes in kidney inflammation and fibrosis in each group of mice, the mRNA expression of inflammatory and fibrosis markers in the kidney tissues was tested. The mRNA expression levels of the inflammation markers *Tnfa*, *Il1b*, and *Il6* in the kidneys of MC mice were 26.86, 5.13, and 3.09 times higher than those in NC mice, respectively. However, the mRNA expression of these three inflammatory markers decreased by 48.97% (*p* = 0.037), 44.66% (*p* = 0.0004), and 58.04% (*p* = 0.0077), respectively, in HTFs mice compared with that in MC mice (Figure 5A–C). In these five groups, the mRNA expression of the fibrosis markers *Tgfb1*, fibronectin, and *ColIV* changed in a similar pattern as the inflammatory marker expression (Figure 5D–F). Comparing the mRNA expression of these inflammatory markers and fibrosis markers in HTFs mice with that in Met mice, no significant differences were found. The protein level changes in TNF-α and TGF-β1 were consistent with changes in their mRNA expression (Figure 5G,H). These data indicated that TFs inhibited the development of inflammation and fibrosis in the kidney tissues of T2DM mice.

### 3.6. TFs May Improve Diabetic Nephropathy through the MAPK/NF-κB Signaling Pathway

**Figure 6:**
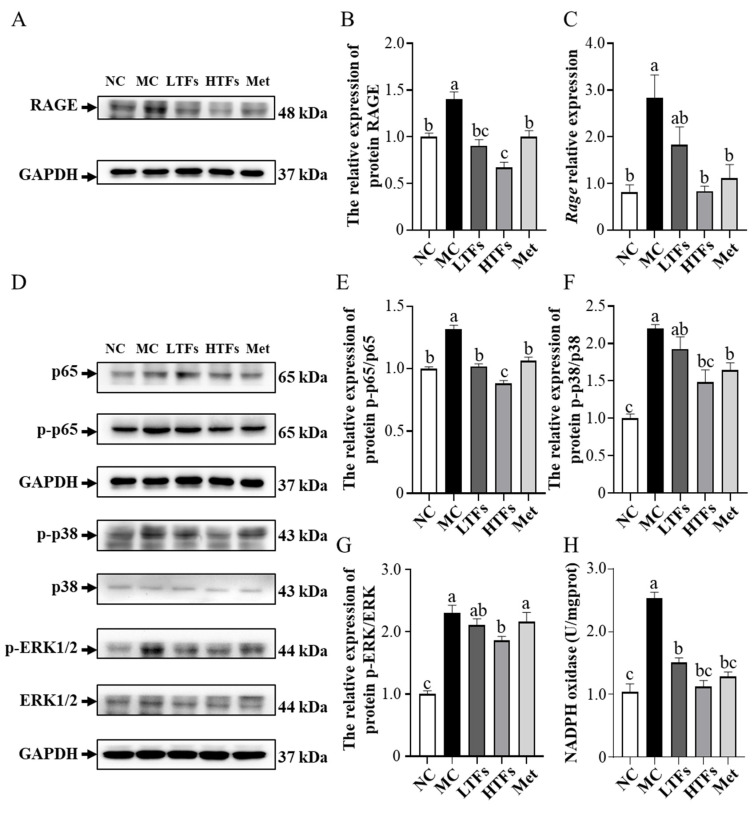
TFs regulate MAPK/NF-κB signal pathway in the kidney tissues of T2DM mice. (**A**) Western blot analysis of RAGE; (**B**) quantification of RAGE protein expression; (**C**) the mRNA expression of RAGE; (**D**) Western blot analysis of MAPK/NF-κB signal pathway, including p65 (**E**), p38 (**F**), ERK1/2 (**G**); (**H**) NADPH oxidase activity. NC: normal control; MC: diabetic model control; LTFs: low-dose TFs; HTFs: high-dose TFs; Met: metformin. Each value represents the mean ± SEM (*n* = 6). Values with different letters (a–c) differ from each other significantly (*p* < 0.05).

As described above, RAGEs were shown to be associated with the activation of the NF-κB and MAPK pathway, responsible for maintaining and amplifying signals, and subsequently inducing inflammatory or fibrotic responses [3,36]. In this study, the protein level of RAGE was found to be 39.90% higher in the kidney tissues of MC mice than in those of NC mice (*p* = 0.007) (Figure 6A,B). After 10 weeks of intervention, the protein level of RAGE in the kidney tissues of LTFs, HTFs, and Met mice decreased by 35.34%, 52.08%, and 28.45%, respectively, compared with that in MC mice (LTFs vs. MC, *p* = 0.0015; HTFs vs. MC, *p* < 0.0001; Met vs. MC, *p* = 0.0071) (Figure 6A,B). This was consistent with the change in the mRNA expression of RAGE (Figure 6C). Further analysis showed that the p65 protein levels in the kidney tissues of mice from each group showed no changes, but the p-p65/p65 ratio in the kidney tissues of MC mice increased to 1.32 times that in NC mice (*p* < 0.0001) (Figure 6D,E). However, the ratio declined significantly after the 10-week TFs or metformin intervention (LTFs vs. MC, *p* < 0.0001; HTFs vs. MC, *p* < 0.0001; Met vs. MC, *p* < 0.0001) (Figure 6D,E). The p38 and ERK1/2 protein expression in the renal tissues of MC mice showed no change compared with that in NC mice, but the protein expression of p-p38 and p-ERK1/2 increased significantly (Figure 6D,G,F). However, after 10 weeks of high-dose TFs or Met intervention, the protein expression of p-p38 MAPK and p-ERK 1/2 decreased significantly (Figure 6D), which suggested that TFs could inhibit the activation of the MAPK/NF-κB signaling pathway in the kidney tissues of diabetic mice. AGEs and RAGE activate the MAPK/NF-κB signaling pathway by altering the NADPH oxidase activity [16,37]. As shown in Figure 6H, the NADPH oxidase activity in the kidneys of MC mice was elevated by 144.23% compared with that in NC mice (NC vs. MC, *p* < 0.0001). After 10 weeks of TFs or metformin intervention, the NADPH oxidase activities in the kidneys of LTFs, HTFs, and Met mice were reduced by 40.47%, 55.55%, and 49.17%, respectively, compared with that in MC mice (LTFs vs. MC, *p* < 0.0001; HTFs vs. MC, *p* < 0.0001; Met vs. MC, *p* < 0.0001). This indicated that TFs could block the MAPK/NF-κB signaling pathway by inhibiting RAGE expression in the kidney tissues of T2DM mice. The lower protein level of RAGE (Figure 6B), lower p-p65/p65 ratio (Figure 6E) and lower p-ERK/ERK ratio (Figure 6G) in the kidney tissues of HTFs mice were observed as compared to those of Met mice, implying that high-dose TFs could work more effectively than metformin.

## 4. Discussion

In the present study, mice fed a high-fat diet and injected with STZ showed symptoms of T2DM, such as weight loss, elevated fasting blood glucose, impaired insulin homeostasis, and others (Figure 1 and Figure 2). These findings were consistent with those observed in a previous mouse model of T2DM established using the same method [38]. After the 10-week intervention with TFs in T2DM mice, high doses of TFs were found to not only significantly reduce the blood glucose level but also improve impaired glucose tolerance and insulin resistance (Figure 2 & Table 1). These findings were consistent with the results in published studies [10,39,40,41,42]. The significant ameliorative effect of TFs on glucose and insulin homeostasis in T2DM mice may be attributed to their ability to restore insulin sensitivity (Table 1).

Diabetic nephropathy is a common complication of T2DM, and its pathological characteristics include persistent proteinuria, altered glomerular filtration rate, glomerular basement membrane thickening, glomerular mesangial expansion, and others [4,43]. In this study, both 24 h urinary protein and urinary albumin levels were more than 10-fold higher in diabetic mice (MC) compared to those in normal controls (NC) (Figure 3A,B). This confirmed diabetic nephropathy [44]. High levels of UCr, SCr, Bun, and GFR indicated the severely impaired glomerular filtration potential in diabetic mice (MC) (Figure 3C–F), which was similar to the findings of Sarkar et al. [11] and Mohan et al. [31]. Additionally, images of HE-stained paraffin sections showed various pathological changes, such as glomerular mesangial expansion, glomerular hyaline, glomerular atrophy, and others, in the kidney tissue of diabetic mice (MC) (Figure 4C,D). However, after 10 weeks of TF intervention, the 24 h urinary protein, urinary albumin, SCr level, Bun level, and GFR of diabetic mice were significantly reduced (Figure 3), and the structural damage also improved significantly (Figure 4). These results indicated that TFs could improve T2DM-induced kidney dysfunction.

As described in the introduction, AGEs are key factors in the development of diabetic nephropathy. Persistent hyperglycemia in patients with diabetes increases the levels of MGO and MDA, which are the precursors of AGEs, which eventually accelerate the synthesis of AGEs [15]. Additionally, the impaired renal function of patients with T2DM prevents the clearance of AGEs by kidneys [3,45]. Numerous AGEs circulate in the blood and accumulate in kidney tissues [46], eventually compromising the renal structure and function [47]. The levels of total AGEs (Table 2), CML and CEL (the major components of AGEs (Table 2)), and HbA1c (an early glycosylation product (Figure 2C)) were significantly elevated in the plasma and kidney tissues of diabetic mice (MC). After 10 weeks of low- or high-dose TFs intervention, the levels of these components decreased significantly. The changes in HOMA-IR and HOMA-IS of LTFs and HTFs group mice suggested that TFs improved the insulin sensitivity in diabetic mice (Table 1), which reduced their blood glucose levels (Figure 2). The lower level of blood glucose in LTFs and HTFs mice led to lesser AGE precursor (MGO and MDA) production compared with that in diabetic mice (Table 2). Eventually, this led to lower AGEs production. In addition, TFs have been shown to scavenge MGO in vitro [6,21,22], thus, TFs may also inhibit the formation of AGEs by scavenging MGO.

The expression of the inflammatory cytokines *Tnfa*, *Il1b*, and *Il6* and fibrosis markers *Tgfb1*, fibronectin, and *ColIV* (Figure 5) was significantly higher in the kidney tissues of diabetic mice (MC) than that in those of normal mice (NC). These abnormally elevated levels of inflammatory cytokines and fibrosis markers suggested that the impairment of renal function and structure might be caused by inflammation and fibrosis in the kidney tissue. The main pathway by which AGEs induce kidney inflammation and fibrosis in diabetic mice is RAGE-mediated MAPK and NF-κB signaling activation [3]. Though the inhibitory effects of TFs on the MAPK and NF-κB pathway activated by AGEs have rarely been reported, Zhu et al. [18] showed that (+)-catechin, a theaflavin precursor, can block cellular ERK1/2- and NF-κB-mediated inflammatory pathways by inhibiting the high glucose-induced elevation of intracellular MGO concentrations in diabetic mice. Meanwhile, Rasheed et al. [48] showed that catechins inhibit AGE-induced intracellular inflammatory responses in chondrocytes by inhibiting p38 and NF-κB activation. As oxidation products of catechins, TFs can protect kidney health in diabetic mice via the MAPK and NF-κB pathway. In this study, the RAGE protein and mRNA levels (Figure 6A–C) as well as the phosphorylation levels of NF-κB p65 in diabetic mice were significantly reduced after TFs intervention (Figure 6D,E). The lower level of AGEs in the TFs intervention mice (Table 2) corresponded to the lower RAGE expression. Li et al. [49] demonstrated that the inhibition of the RAGE-mediated phosphorylation of NF-κB p65 can help resist chronic inflammation in renal tissues. The low RAGE level prevented the activation of NF-κB in the kidney tissues of diabetic mice, which was confirmed by changes in the mRNA expression of NF-κB downstream genes, such as *Tnfa*, *Il1b*, and *Il6* (Figure 5). However, AGEs and RAGE do not act directly on NF-κB. AGEs bind to RAGE and increase NADPH oxidase activity which initiates ERK l/2 and p38 [50,51]. Subsequently, NF-κB is activated by MAPK signaling, which triggers inflammation and fibrosis [51]. In the present study, the activity of NADPH oxidase (Figure 6H) and levels of p-p38 and p-ERK1/2 (Figure 6D) were significantly increased in the kidney tissues of diabetic mice (MC) compared with normal mice (NC), but the levels were significantly reduced by high-dose TFs intervention (Figure 6F–H). Compared with that in diabetic mice, the lower activity of NADPH oxidase in the kidney tissues of high-dose TFs intervention mice was owing to fewer AGEs and RAGE molecules. The lower NADPH oxidase activity resulted in lower levels of p-p38, p-ERK1/2 and p-p65, which eventually inhibited inflammation and fibrosis in the kidney tissues of mice subjected to high-dose TFs intervention.

## 5. Conclusions

**Figure 7:**
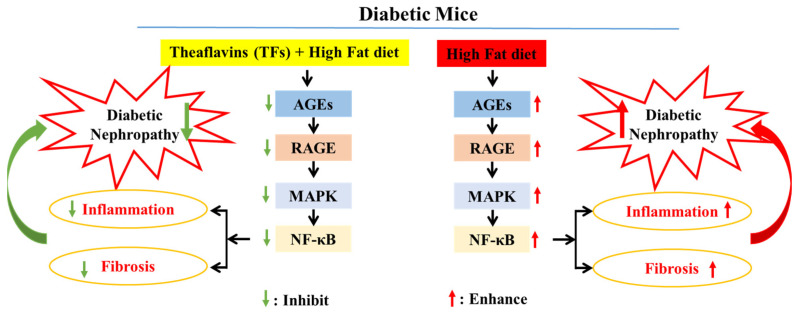
Potential mechanism of kidney injury mediated by AGEs in T2DM mice treated with TFs.

The persistent hyperglycemia in diabetic mice contributed to the high levels of MGO and MDA in vivo, thereby exacerbating the formation of AGEs. AGEs bind to RAGE and boost NADPH oxidase activity, which activates the MAPK/NF-κB signaling pathway. Subsequently, the activated MAPK/NF-κB signaling pathway triggers inflammation and fibrosis in the kidney tissues of diabetic mice, eventually leading to diabetic nephropathy (Figure 7). In this study, TFs improved blood glucose levels and inhibited the formation of AGEs, which reduced the expression of RAGE. Subsequently, AGEs, present at low levels, bound to RAGE and prevented inflammation and fibrosis by inhibiting the MAPK/NF-κB signaling pathway, eventually improved diabetic nephropathy.

## Figures and Tables

**Table 1:** Changes in HOMA-IR and HOMA-IS of T2DM mice.

Parameter	NC	MC	LTFs	HTFs	Met
Insulin (mU/L)	20.58 ± 1.95 ^c^	49.68 ± 2.56 ^a^	39.82 ± 3.01 ^b^	25.87 ± 3.90 ^c^	21.43 ± 4.08 ^c^
HOMA-IR	6.03 ± 0.72 ^c^	59.64 ± 10.85 ^a^	32.16 ± 8.62 ^b^	21.36 ± 7.02 ^bc^	12.03 ± 2.07 ^c^
HOMA-IS	0.17 ± 0.022 ^a^	0.017 ± 0.0036 ^d^	0.033 ± 0.0082 ^cd^	0.051 ± 0.016 ^c^	0.085 ± 0.015 ^b^

NC: normal control; MC: diabetic model control; LTFs: low-dose TFs; HTFs: high-dose TFs; Met: metformin. Each value represents the mean ± SEM (*n* = 6). Values at the same row with different letters (a−d) differ from each other significantly (*p* < 0.05).

**Table 2:** Changes in AGEs and their precursor substances in T2DM mice.

Parameter	NC	MC	LTFs	HTFs	Met
Plasma					
Total AGEs (AU)	56.50 ± 1.67 ^b^	80.03 ± 8.56 ^a^	60.26 ± 1.45 ^b^	58.07 ± 1.29 ^b^	59.11 ± 1.19 ^b^
CML (ng/mL)	1252.20 ± 64.86 ^b^	1524.56 ± 80.30 ^a^	1098.20 ± 53.16 ^bc^	990.18 ± 46.48 ^c^	684.49 ± 36.21 ^d^
CEL (ng/mL)	14.00 ± 0.48 ^b^	17.19 ± 0.62 ^a^	15.08 ± 0.50 ^b^	11.17 ± 0.34 ^c^	8.12 ± 0.30 ^d^
MGO (ng/mL)	19.09 ± 0.29 ^b^	21.93 ± 0.43 ^a^	11.29 ± 0.49 ^d^	14.49 ± 0.39 ^c^	13.69 ± 0.83 ^c^
MDA (nmol/mL)	2.53 ± 0.057 ^d^	7.21 ± 0.11 ^a^	5.51 ± 0.25 ^b^	4.47 ± 0.20 ^c^	4.84 ± 0.24 ^bc^
Kidney					
Total AGEs (AU/mgprot)	0.17 ± 0.027 ^c^	0.56 ± 0.028 ^a^	0.49 ± 0.031 ^ab^	0.41 ± 0.042 ^b^	0.43 ± 0.019 ^ab^
CML (ng/mgprot)	389.41 ± 33.56 ^a^	460.32 ± 30.35 ^a^	217.28 ± 3.07 ^b^	359.05 ± 22.84 ^a^	359.60 ± 10.53 ^a^
CEL (ng/mgprot)	3.87 ± 0.22 ^bc^	5.42 ± 0.47 ^a^	5.08 ± 0.31 ^ab^	2.88 ± 0.21 ^c^	3.79 ± 0.20 ^c^
MGO (ng/mgprot)	4.46 ± 0.40 ^b^	7.44 ± 0.41 ^a^	6.98 ± 0.48 ^a^	4.83 ± 0.24 ^b^	5.03 ± 0.16 ^ab^
MDA (nmol/mgprot)	0.72 ± 0.061 ^c^	2.20 ± 0.010 ^a^	1.47 ± 0.054 ^b^	1.35 ± 0.015 ^b^	1.22 ± 0.12 ^b^

NC: normal control; MC: diabetic model control; LTFs: low-dose TFs; HTFs: high-dose TFs; Met: metformin. Each value represents the mean ± SEM (*n* = 6). Values at the same row with different letters (a−d) differ from each other significantly (*p* < 0.05).

## Data Availability

Data were available upon reasonable request to the corresponding authors.
